# The effect of exercise training on the course of cardiac troponin T and I levels: three independent training studies

**DOI:** 10.1038/srep18320

**Published:** 2015-12-16

**Authors:** Noreen van der Linden, Lieke J. J. Klinkenberg, Marika Leenders, Michael Tieland, Lex B. Verdijk, Marijke Niens, Jeroen D. E. van Suijlen, Lisette C. P. G. M. de Groot, Otto Bekers, Luc J. C. van Loon, Marja P. van Dieijen-Visser, Steven J. R. Meex

**Affiliations:** 1 Department of Clinical Chemistry, Cardiovascular Research Institute Maastricht (CARIM), Maastricht University Medical Center (MUMC), Maastricht, The Netherlands; 2 Department of Human Movement Sciences, School for Nutrition, Toxicology and Metabolism (NUTRIM), Maastricht University Medical Center (MUMC), Maastricht, The Netherlands; 3 Top Institute Food and Nutrition, Wageningen University, Wageningen, The Netherlands; 4 Division of Human Nutrition, Wageningen University, Wageningen, The Netherlands; 5 Department of Clinical Chemistry, Gelre Ziekenhuizen, Apeldoorn, The Netherlands

## Abstract

With the introduction of high-sensitive assays, cardiac troponins became potential biomarkers for risk stratification and prognostic medicine. Observational studies have reported an inverse association between physical activity and basal cardiac troponin levels. However, causality has never been demonstrated. This study investigated whether basal cardiac troponin concentrations are receptive to lifestyle interventions such as exercise training. Basal high-sensitive cardiac troponin T (cTnT ) and I (cTnI) were monitored in two resistance-type exercise training programs (12-week (study 1) and 24-week (study 2)) in older adults (≥65 years). In addition, a retrospective analysis for high sensitive troponin I in a 24-week exercise controlled trial in (pre)frail older adults was performed (study 3). In total, 91 subjects were included in the final data analyses. There were no significant changes in cardiac troponin levels over time in study 1 and 2 (study 1: cTnT −0.13 (−0.33–+0.08) ng/L/12-weeks, cTnI −0.10 (−0.33–+0.12) ng/L/12-weeks; study 2: cTnT −1.99 (−4.79–+0.81) ng/L/24-weeks, cTnI −1.59 (−5.70–+2.51) ng/L/24-weeks). Neither was there a significant interaction between training and the course of cardiac troponin in study 3 (p = 0.27). In conclusion, this study provides no evidence that prolonged resistance-type exercise training can modulate basal cardiac troponin levels.

The development of increasingly sensitive assays for cardiac troponin has now reached the point where cardiac troponin concentrations can be accurately assessed in the majority of subjects from a healthy reference population^1^. Parallel to the development of assays with increasing sensitivity, interest in cardiac troponin has expanded from acute cardiac care to risk prediction and risk stratification^2^. Studies in various patient groups and asymptomatic individuals have provided compelling evidence that baseline cardiac troponin levels predict outcome^3^. Interestingly, the observed risk gradient is not restricted to elevations above the 99^th^ percentile, but is even apparent within the “healthy” population reference interval, and independent of traditional risk factors such as age, sex and diabetes^4^,^5^,^6^,^7^,^8^,^9^. In the Framingham heart study, the basal cardiac troponin I concentration adds prognostic value to standard risk factors for predicting death, and cardiovascular disease^10^. Furthermore, temporal increases in cardiac troponin concentrations over time confer additional cardiovascular risk, and are inversely related to the level of physical fitness^7^,^11^. These observations form the basis of the hypothesis that cardiac troponin is a modifiable parameter, which may be receptive to lifestyle interventions such as an exercise training program. In a recent study with (pre)frail subjects we tested the hypothesis that a supervised resistance-type exercise training program can influence the course of cardiac troponin levels over time^12^. However, this 24-week supervised bi-weekly exercise training program conferred no beneficial effect on the course of cardiac troponin T levels, despite substantial improvements of these subjects at the level of physical performance^12^. This negative study was conducted in frail and pre-frail subjects, characterized by substantially elevated baseline cardiac troponin levels, and therefore theoretically most amenable to the potential benefits of an exercise intervention. A possible drawback however, was the limited training load that could be imposed due to the level of frailty of these older subjects. Nevertheless, despite these limitations, our results were similar to those of other recent reports: 14-week endurance training in untrained adults^13^, 17-week half-marathon training in previously sedentary men^14^, and 3-month exercise training in heart failure patients (NYHA class II-IV)^15^ did not result reduce basal cardiac troponin T concentrations. However, limitations of these studies were the low basal cardiac troponin concentrations^13^ and the use of conventional instead of high-sensitive cardiac troponin T assays^14^,^15^ which reduced the power to detect an effect. Another issue that merits attention is that the effect of training intervention on cardiac troponin I has never been assessed. Since cardiac troponin T and I are two different proteins of the cardiac troponin complex with different biochemical characteristics^16^ and distinct release patterns^17^, both cardiac troponins may also respond differently to lifestyle interventions.

The aim of the current study is 1) to overcome the limitations of previous studies, and 2) to examine the effect of exercise training on basal cardiac troponin I levels. Therefore, we now present two additional exercise training studies to the effect of a more intense training regimen on the course of high-sensitive cardiac troponin T and I levels in older adults, a population that often exhibits elevated basal cardiac troponin concentrations. Additional high sensitive troponin I was measured in our previously conducted study in (pre)frail subjects in order to present a complete and comprehensive overview to the effects of three supervised training programs.

## Methods

### Study design

To investigate the effect of resistance-type exercise training on cardiac troponin T (cTnT) and I (cTnI) in older adults, two independent studies were conducted: a study to the effect of 12-week resistance-type exercise training in older men (≥65 years) (study 1) and a second study to the effect of a 24-week resistance-type exercise training older men and women (≥65 years) (study 2). In addition, we performed a retrospective analysis for cardiac troponin I in our recently published study to the effect of a 24-week resistance-type exercise training program on the course of troponin T levels in (pre)frail subjects (≥65 years) (study 3).

All studies complied with the principles of the Declaration of Helsinki and were approved by the local institutional review boards and the ethics committees of Wageningen University or Maastricht University Medical Center. Participants were recruited by advertisements in local newspapers (study 1 and 2) or by approaching elderly living in apartment houses and in homes for the elderly (study 3). All participants provided written informed consent. Subjects were only eligible for inclusion when they had not participated in any structured exercise training program over the past 2 years.

### Study 1: 12-week training in older men

In total 14 subjects were included in study 1. Inclusion criteria were age (65–85 years), male gender and the ability to understand and perform study procedures. Exclusion criteria were (silent) cardiac or peripheral vascular disease, orthopedic limitations (self-reported) and type 2 diabetes (based on an oral glucose tolerance test). A training session consisted of a 5-minute warming up on a cycle ergometer, four sets on leg-press and leg-extension machines and a 5-minute cooling down on the cycle ergometer. The workload started at 60% of 1 repetition maximum (1-RM) (10–15 repetitions per set) and was increased to 75–80% of 1-RM (8–10 repetitions per set). The typical duration of a training session was approximately 45 minutes, and the work:rest ratio was 1:4. This supervised resistance-type exercise training program was performed three times per week during 12 weeks. There was at least a resting period of 2 days between consecutive training sessions. Blood samples were obtained by a venipuncture after an overnight fast, at least three days after the last training session, before the start of the training program (week 0) and after 4, 8 and 12 weeks of intervention. This study is a substudy of a more extensive trial examining the effects of resistance-type exercise training in older men on muscle strength, body composition and muscle fiber type-specific characteristics as primary outcome^18^.

### Study 2: 24-week training in older men and women

In total 27 subjects were included in study 2. Inclusion criteria were age (65–90 years) and the ability to understand and perform study procedures. Exclusion criteria were the same as in study 1. Training sessions consisted of a 5-minute warming up on a cycle ergometer, four sets on leg-press and leg-extension machines and three sets on the chest press and horizontal row; these four exercises were performed every training session. In addition, three sets of vertical lat pull and abdominal crunches were alternated with biceps curl and triceps extension between subsequent training sessions. The workload started at 60% of 1-RM (10–15 repetitions per set) and was increased to 75–80% of 1-RM (8–10 repetitions per set). The typical duration of a training session was approximately 45 minutes, and the work:rest ratio was 1:4. This supervised resistance-type exercise training program was performed three times per week during 24 weeks. There was at least a resting period of 2 days between consecutive training sessions. Blood samples were obtained by a venipuncture after an overnight fast at least three days after the last training session before the start of the training program (week 0) and after 4, 8, 12, 16, 20 and 24 weeks of intervention. This is a substudy of a more extensive trial to the effects of an exercise training intervention in older adults on muscle strength, body composition and muscle fiber type-specific characteristics. In this current study we included only subjects from the control group who were not receiving dietary protein supplementation (as described in the original study protocol)^19^,^20^.

### Study 3: 24-week training in (pre)frail older men and women

Methodological details of study 3 have been described previously^12^. Briefly, a total of 62 (pre)frail older men and women (≥65 years) were included and were equally distributed in an intervention and a control group. Frailty and prefrailty were defined according to the Fried criteria (subjects were considered prefrail when 1 or 2 criteria were applicable, and frail when 3 or more criteria were present): (1) unintentional weight loss, (2) weakness, (3) self-reported exhaustion, (4) slow walking speed, and (5) low physical activity^21^. The intervention group participated in a 24-week, biweekly, supervised resistance-type exercise training program. Blood samples were obtained by a venipuncture after an overnight fast before the start of the training program (week 0), after 12 and after 24 weeks of intervention. The effect of the training program on muscle mass, muscle strength, physical performance, and troponin T levels were published recently. In this current study we included only subjects from the control group who were not receiving dietary protein supplementation (as described in the original study protocol)^12^,^22^,^23^.

### Cardiac troponin T and I

Plasma and serum samples were stored at −80 °C until analysis. Serum (study 1) and plasma (study 2 and 3) cardiac troponins were measured using the high sensitive cTnT assay (limit of blank 3.0 ng/L, limit of detection 5.0 ng/L, 99th percentile 14 ng/L^24^) on the Cobas analyzer (Roche Diagnostics) and the high sensitive cTnI assay (according to the manufacturer, limit of blank 0.7–1.3 ng/L, limit of detection 1.1–1.9 ng/L, 99^th^ percentile 26.2 ng/L and 10% CV 4.7 ng/L) on the ARCHITECT analyzer (Abbott Diagnostics).

### Statistical analysis

Normally distributed parameters were expressed as mean ± standard deviation (SD), non-normally distributed parameters as median and interquartile range (IQR), and categorical variables as number (n) and percentage (%). Changes in basal cardiac troponin T and I concentrations over time were analyzed using mixed linear model analyses (random intercept) with the covariate time as fixed effect. The inclusion of 14 subjects in study 1 and 27 subjects in study 2 afforded at least 90% power at a significance level of 5% to detect a 10% reduction in cardiac troponin levels, anticipating a dropout rate of 25%. These post-hoc power calculations were based on the assumption of a mean baseline cTnT concentration of 8.0 ng/L and cTnI concentration of 6.0 ng/L for study 1 and a mean baseline cardiac cTnT concentration of 6.0 ng/L and a cTnI concentration of 4.0 ng/L for study 2 with older men and women (estimated baseline correlation and decay rate 0.90 and 0.25, respectively)^25^. We used two-level design mixed linear model analyses (random intercept) with time and intervention as fixed effects to assess the differences in the course of cardiac troponin I between (pre)frail subjects in the control group and in the intervention group. Power calculation for study 3 has been described previously^12^. All statistical analyses were performed using SPSS, version 20.0. P ≤ 0.05 was considered statistically significant for all analyses.

## Results

### Compliance to the training program

Included subjects in study 1 and 2 demonstrated 97% and 90% compliance, respectively, to the scheduled, supervised training sessions. In each study one subject withdrew. Ten subjects (15%) withdrew from study 3. Subjects withdrew because of time constraints and medical reasons unrelated to the exercise training. In total, in the final data analysis we included: 13 older men participating in the 12-week training program (study 1), 26 older men and women participating in the 24-week training program (study 2), and 52 subjects in the retrospective troponin I analysis with (pre)frail older men and women (study 3). Table 1 shows the subjects’ clinical characteristics at baseline. Baseline characteristics for the (pre)frail subjects have been published previously^12^.

### No decrease in cardiac troponin T and I levels during the course of the exercise training program

**Figure 1:**
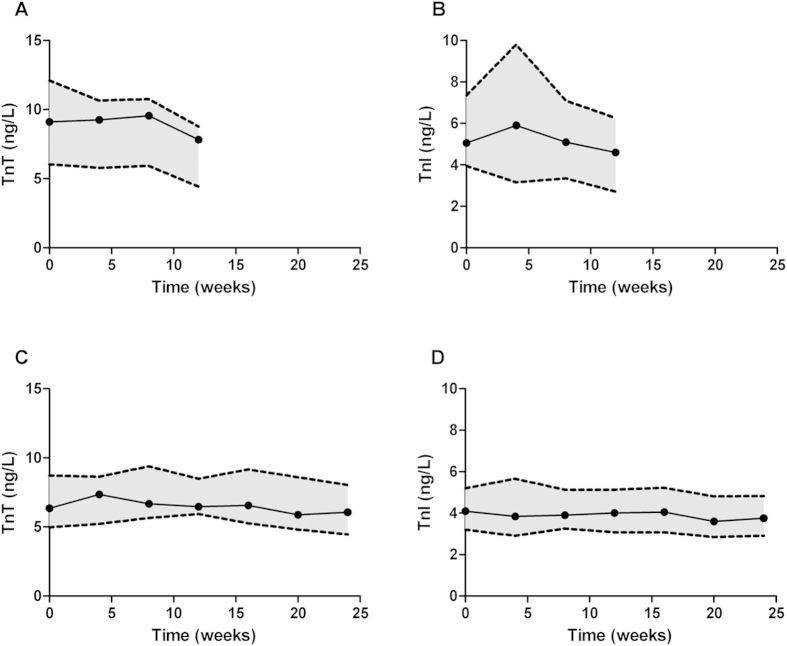
Course of cardiac troponinT and I in older adults participating in 12- and 24-week training programs. Median (interquartile range) concentrations of cTn in older adults participating in a supervised resistance-type training program: (**A**) course of cTnT in older men in a 12-week training program; (**B**) course of cTnI in older men in a 12-week training program; (**C**) course of cTnT in older men and women in a 24-week training program; (**D**) course of cTnI in older men and women in a 24-week training program.

Figure 1 shows the course of cardiac troponin T and cardiac troponin I levels over time in study 1 and 2 (for individual data see Supplementary Fig. S1 and S2 online). Table 2 shows the corresponding baseline and the follow-up values for cTnT and cTnI. One subject in study 2 (3% of the participants in study 1 and 2) had cardiac troponin T concentrations below the limit of blank (3ng/L) at all time points, and was therefore excluded from the cardiac troponin T analysis. In study 1 and 2 98.6% and 99.6% of all other troponin T and I measurements respectively had concentrations above the limit of blank. Using mixed linear model analyses, we found no significant changes in cardiac troponin levels over time in older men participating in a 12-week resistance-type exercise training program (cTnT −0.13 (−0.33–+0.08) ng/L/12-weeks (p = 0.16), cTnI −0.10 (−0.33–+0.12) ng/L/12-weeks (p = 0.44)) and in older men and women participating in a 24-week resistance-type exercise training program (cTnT −1.99 (−4.79–+0.81) ng/L/24-weeks (p = 0.23), cTnI −1.59 (−5.70–+2.51) ng/L/24-weeks (p = 0.37)).

### No effect of a 24-week resistance-type exercise training program on cardiac troponin I levels in (pre)frail older adults

Table 3 shows the baseline cTnI concentrations and the 12-week and 24-week follow-up data of (pre)frail older men and women. In the control group, all values were above the limit of blank of the assay (1.3 ng/L), 3 subjects had at least one value above the 99^th^ percentile of 26.2 ng/L, which is used for the diagnosis of myocardial infarction. In the intervention group, one subject had one value below limit of blank of the assay, and one subject had at least one value above the 99^th^ percentile. Mixed linear model analyses revealed no significant interaction between training and the course of cTnI levels between the intervention and the control group (intention-to-treat analysis p = 0.27, per-protocol analysis p = 0.27) (for individual data see Supplementary Fig. S2 online).

## Discussion

In two independent supervised training studies of 12 and 24 weeks respectively, we found no effect of resistance-type exercise training on the course of cardiac troponin T and I levels in older subjects, nor did we find an effect of a 24-week training program on cardiac troponin I levels in (pre)frail subjects.

Our results do not support the idea that cardiac troponin levels might be modifiable through exercise. This hypothesis was postulated by observational studies, showing that higher physical activity levels in older subjects were associated with both lower basal cTnT levels, and a lower probability of a significant increase in cTnT concentrations between consecutive visits^11^,^26^. To investigate whether a causal relationship underlies this association, we conducted a series of training studies in subjects where stable elevated cardiac troponin levels are common, and who are therefore -at least theoretically- most receptive to the favorable effects of an intervention. To ensure that basal cardiac troponin levels, rather than acute post-exercise effects were studied, we included a three day interval between the last training session and the blood sampling procedure^27^. None of these studies showed a favorable effect of resistance-type exercise training on cardiac troponin levels. The non-responsive pattern of troponin T and I to an exercise program is in contrast with the beneficial changes observed for leg strength, muscle mass, insulin sensitivity, HbA1c, total cholesterol and LDL^18^,^19^,^20^,^22^,^23^. The present results confirm and extend the results of our previous training intervention study in (pre)frail older adults^12^, which was also characterized by favorable effects on physical performance, but a lack of effect on circulating cardiac troponin levels.

Although the absolute number of subjects in these training studies are relatively low, all studies afforded at least 90% power to detect a 10% reduction of cardiac troponin over time. We cannot exclude the possibility that training confers a smaller effect on the course of troponin levels that goes by undetected in the present study, but we feel that (much) smaller changes than specified in our power calculation would comprise limited clinical relevance. A possible limitation of our study relates to the type of exercise training intervention, comprising mostly resistance-type exercise. However the subjects’ physical state precluded (intense) endurance training. Despite the physical and metabolic improvements found in our studies^18^,^19^,^20^,^22^,^23^, a valid question may be whether resistance-type exercise training is the most appropriate type of training to generate a favorable effect on cardiac troponin concentrations. In this respect the results of a recently conducted cardio-based exercise training intervention study in heart failure patients are interesting. Similar to our studies, no favorable effect was found of a supervised endurance exercise training program on cardiac troponin T levels^15^.

The supervision during the sessions contributed to the internal validity, but might also have led to increased safety, less adverse events, and a higher motivation among subjects. This may overestimate any beneficial effects of exercise training. Another point regarding the validity of our study is the participant recruitment process and study design. Since participation in the study is associated with a substantial time investment and a relatively high intensity training program, this might have attracted a specific, highly motivated subpopulation. Since our study was conducted in an elderly population we can only speculate about the effects of exercise training on basal cardiac troponin levels in younger subjects who may respond differently on exercise training and demonstrate higher cardiovascular plasticity^28^. Nevertheless, our study does not provide support for the hypothesis that basal cardiac troponin concentrations are receptive to a prolonged resistance-type exercise training program.

## Additional Information

**How to cite this article**: van der Linden, N. *et al.* The effect of exercise training on the course of cardiac troponin T and I levels: three independent training studies. *Sci. Rep.* **5**, 18320; doi: 10.1038/srep18320 (2015).

## Supplementary Material

Supplementary Information

## Figures and Tables

**Table 1:** Clinical Characteristics.

Variable	Older men 12-w intervention (n = 13)	Older men and women 24-w intervention (n = 26)	(pre)Frail older men and women 24-w intervention (n = 25)	(pre)Frail older men and women control (n = 27)
Demographics				
Age (years), mean (SD)	72.4 (5.4)	69.2 (4.1)	78.6 (6.8)	80.2 (7.4)
Women, n (%)	0 (0)	12 (46)	17 (68)	14 (52)
BMI (kg/m^2^), mean (SD)	27.4 (3.8)	25.9 (2.0)	28.7 (4.9)	26.6 (3.3)
Medical history				
Systolic BP (mm Hg), mean (SD)	147 (14)	137 (15)	142 (18)	151 (24)
Diastolic BP (mm Hg), mean (SD)	82 (17)	77 (22)	72 (9)	75 (9)
Laboratory data				
Fasting glucose (mmol/L), mean (SD)	5.6 (0.7)	5.5 (0.5)	5.3 (0.5)	5.2 (0.5)
Total cholesterol (mmol/L), mean (SD)	5.6 (1.1)	6.2 (0.9)	5.2 (1.5)	5.4 (1.1)
Triglycerides (mmol/L), mean (SD)	1.4 (0.5)	1.3 (0.5)	1.3 (0.5)	1.2 (0.6)
HDL cholesterol (mmol/L), mean (SD)	1.3 (0.4)	1.6 (0.3)	1.3 (0.3)	1.5 (0.4)
LDL cholesterol (mmol/L), mean (SD)	3.6 (1.3)	4.3 (0.8)	3.2 (1.3)	3.4 (0.9)
Creatinine (μmol/L), mean (SD)	102.2 (7.5)	88.1 (17.4)	74.4 (14.1)	77.7 (12.1)
Cystatin C (mg/L), mean (SD)	1.07 (0.11)	0.90 (0.19)	1.00 (0.29)	1.08 (0.25)
eGFR (ml/min/1.73 m^2^), mean (SD)*	66.3 (7.2)	78.3 (15.2)	73.9 (15.4)	69.6 (13.9)
NT-proBNP (pg/mL), median (IQR)	12.1 (6.8–21.0)	7.6 (3.1–15.8)	14.6 (6.2–25.3)	19.1 (8.0–46.3)
Medication use				
Antihypertensives, n (%)	3 (25)	3 (12)	11 (44)	14 (52)
Statins, n (%)	1 (8)	1 (4)	9 (36)	3 (11)

*eGFR, estimated glomerular filtration rate based on the the CKD-EPI creatinine-cystatin C equation (2012)^29^.

**Table 2:** Baseline and follow-up concentrationsof cTn in older adults participating in 12-week and 24-week training programs.

	hs-cTnT (ng/L) ), median (IQR)		hs-cTnI (ng/L) ), median (IQR)	
	12-w intervention (n = 13)	24-w intervention (n = 25)	12-w intervention (n = 13)	24-w intervention (n = 26)
**Baseline**	9.1 (6.0–12.1)	6.4 (5.0–8.7)	5.1 (4.0–7.4)	4.1 (3.2–5.2)
**Week 4**	9.3 (5.8–10.7)	7.4 (5.2–8.6)	5.9 (3.2–9.8)	3.9 (2.9–5.7)
**Week 8**	9.6 (5.9–10.8)	6.7 (5.6–9.4)	5.1 (3.4–7.1)	3.9 (3.3–5.1)
**Week 12**	7.8 (4.4–8.8)	6.5 (5.9–8.5)	4.6 (2.7–6.3)	4.0 (3.1–5.1)
**Week 16**		6.6 (5.3–9.2)		4.1 (3.1–5.2)
**Week 20**		5.9 (4.8–8.6)		3.6 (2.9–4.8)
**Week 24**		6.1 (4.5–8.0)		3.8 (2.9–4.8)

**Table 3:** Baseline and follow-up concentrations of hs-cTnI in (pre)frail older subjects in the control group and those participating in a 24-week resistance-type exercise training program.

Variable	(pre)Frail older subjects 24-w intervention (n = 25)	(pre)Frail older subjects no intervention (n = 27)
Baseline hs-cTnI (ng/L), median (IQR)	4.4 (3.3–6.2)	6.7 (4.7–7.7)
12-week hs-cTnI (ng/L), median (IQR)	4.6 (3.5–6.4)	6.7 (4.4–8.4)
24-week hs-cTnI (ng/L), median (IQR)	4.3 (3.6–8.4)	6.0 (3.4–7.6)
