# Supplementary material for: The effect of exercise training on the course of cardiac troponin T and I levels: three independent training studies
Source: Sci Rep. 2015 Dec 16;5:18320. doi: 10.1038/srep18320 (PMC4680870; doi:10.1038/srep18320)
Supplement: Supplementary Information [file srep18320-s1.doc]

**Supplementary Information**

**The effect of exercise training on the course of cardiac troponin T and I levels: three independent training studies.**

Noreen van der Linden^1^, Lieke J.J. Klinkenberg^1^, Marika Leenders^2,3^, Michael Tieland^3,4^, Lex B. Verdijk^2,3^, Marijke Niens^5^, Jeroen D.E. van Suijlen^5^, Lisette C.P.G.M. de Groot^3,4^, Otto Bekers^1^, Luc J.C. van Loon^2,3^, Marja P. van Dieijen-Visser^1^, Steven J.R. Meex^1^*

*^1^ Department of Clinical Chemistry, Cardiovascular Research Institute Maastricht (CARIM), Maastricht University Medical Center (MUMC), Maastricht, The Netherlands.*

*^2^ Department of Human Movement Sciences, School for Nutrition, Toxicology and Metabolism (NUTRIM), Maastricht University Medical Center (MUMC), Maastricht, The Netherlands.*

*^3^ Top Institute Food and Nutrition, Wageningen University, Wageningen, The Netherlands*

*^4^ Division of Human Nutrition, Wageningen University, Wageningen, The Netherlands.*

*^5^ Department of Clinical Chemistry, Gelre Ziekenhuizen, Apeldoorn, The Netherlands.*

**Supplementary Figure S1.** Individual troponin T profiles

**Supplementary Figure S2.** Individual troponin I profiles

**Supplementary Figure S1. Individual troponin T profiles**

**Supplementary Figure S2. Individual troponin I profiles**
